# Supplementary material for: Encapsidation of Different Plasmonic Gold Nanoparticles by the CCMV CP
Source: Molecules. 2020 Jun 5;25(11):2628. doi: 10.3390/molecules25112628 (PMC7321416; doi:10.3390/molecules25112628)
Supplement: Supplementary file 1 [file molecules-25-02628-s001.pdf]

Supplementary Material

Encapsidation of Different Plasmonic Gold Nanoparticles by the CCMV Capsid Protein

Ana L. Durán-Meza [1], Martha I. Escamilla-Ruiz [1], Xochitl F. Segovia-González [1], Maria V. Villagrana-Escareño [1], J. Roger Vega-Acosta [1] and Jaime Ruiz-Garcia [1,*]

[1] Biological Physics Laboratory, Universidad Autónoma de San Luis Potosí, Álvaro Obregón 64, San Luis Potosí, S. L. P., 78000 México; analuisa.duranmeza@gmail.com (A.L.D-M); iggy_27@hotmail.com (M.I.E-R); xochitl.guao@gmail.com (X.F.S-G); veronica.villagrana@gmail.com (M.V.V-E); rogerveg@mail.ifisica.uaslp.mx (J.R.V-A);

* Correspondence: jaime@mail.ifisica.uaslp.mx; Tel.: 011-52-444-8262362

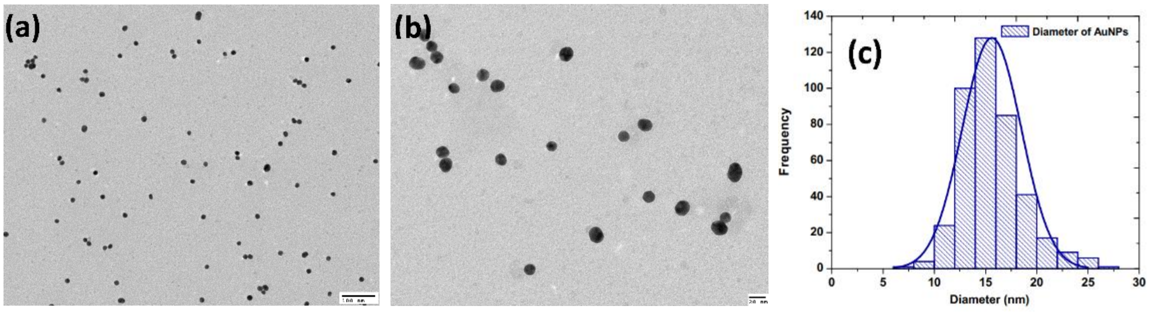

S1. Transmission Electron Microscopy (TEM) of gold nanoparticles of an average of 18 nm (hydrodynamic diameter obtained by DLS) at different scale a). The scale bar is of 100 nm, and b) the scale bar is of 20 nm. C). Corresponds to diameter size distribution of these nanoparticles obtained from TEM images using ImageJ.

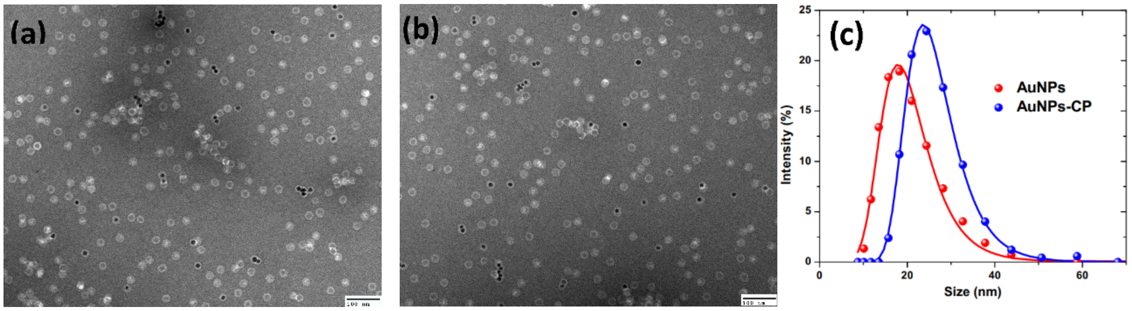

S2. Transmission Electron Microscopy (TEM) images of gold Nanoparticles encapsidated by capsid protein of CCMV. The scale bar in figs. a) and b) is 100 nm. We can observe the formation of a large number of empty capsids due to an excess of protein to secure that all AuNPs get encapsidated. c).This image shows a comparison between naked (red) and encapsidated AuNPs (blue). The difference on the average size, corresponds to twice the thickness of the protein capsid

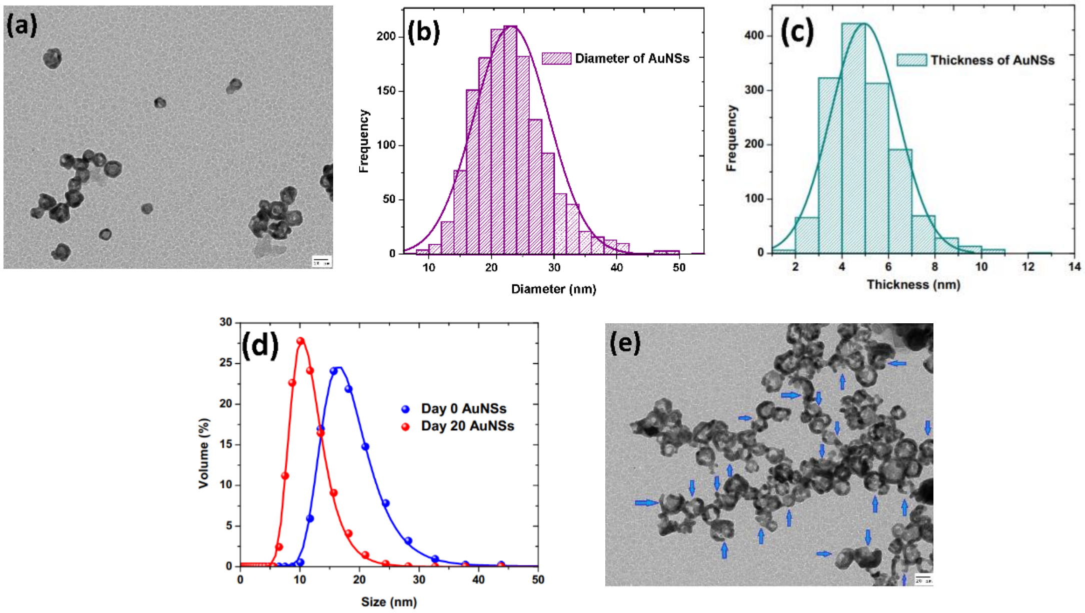

S3. a). TEM images of gold nanoshells the scale bar is 20 nm. b). Diameter size distribution of AuNSs, c). Thickness of AuNSs using TEM images. The analysis of the images to obtain Figs. b) and c) was performed using Image J, analyzing two hundred particles in two directions. d). Histogram on the stability of the AuNSs as a function of time obtained by DLS. The blue line corresponds to the AuNSs obtained on the day they were synthesized. The red line corresponds to the size of the AuNSs 20 days after the synthesis. It is clear how the size decreases due to the Ostwald ripening process. e) The arrows show broken gold nanoshells after 20 days due to Ostwald ripening.

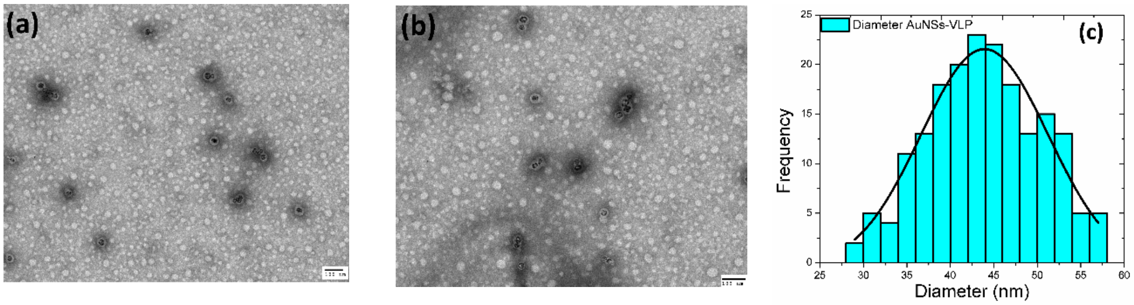

S4. a) and b). TEM images of encapsidated AuNSs by capsid protein of CCMV virus. The scale bar is 100 nm. We can observe that there are a large number of empty capsids, but all gold nanoshells are encapsidated. c). Size distribution of the encapsidated AuNSs obtained from the TEM images after analyzing 200 particles in two directions.

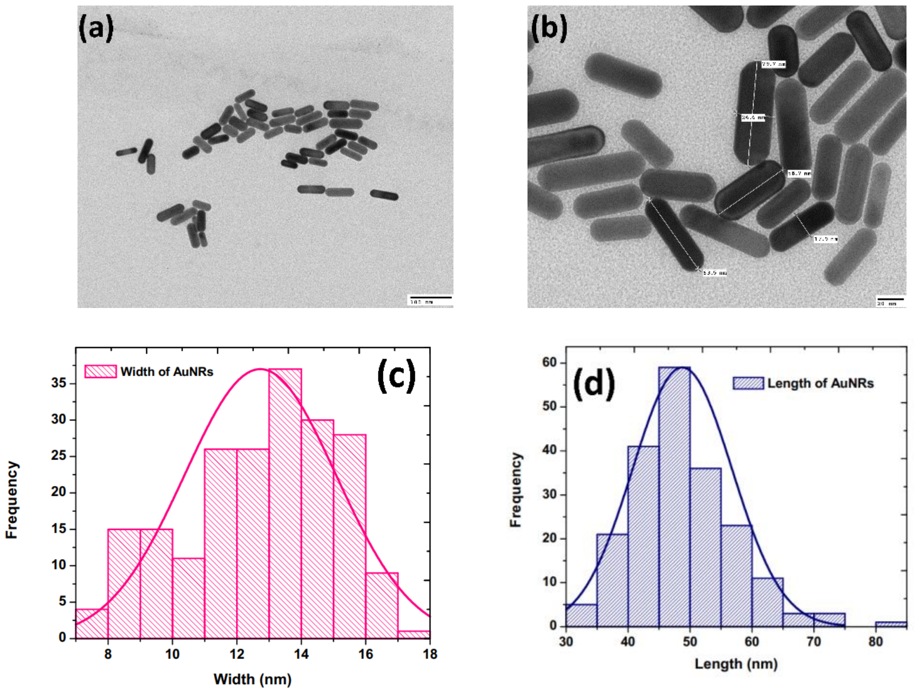

S5. a) and b). TEM images of gold nanorods the scale bar in a) is 100 nm and in b) is 20 nm. c). Width distribution of AuNRs d) Length distribution of AuNRs, analyzing 200 AuNRs in two positions for each graph.

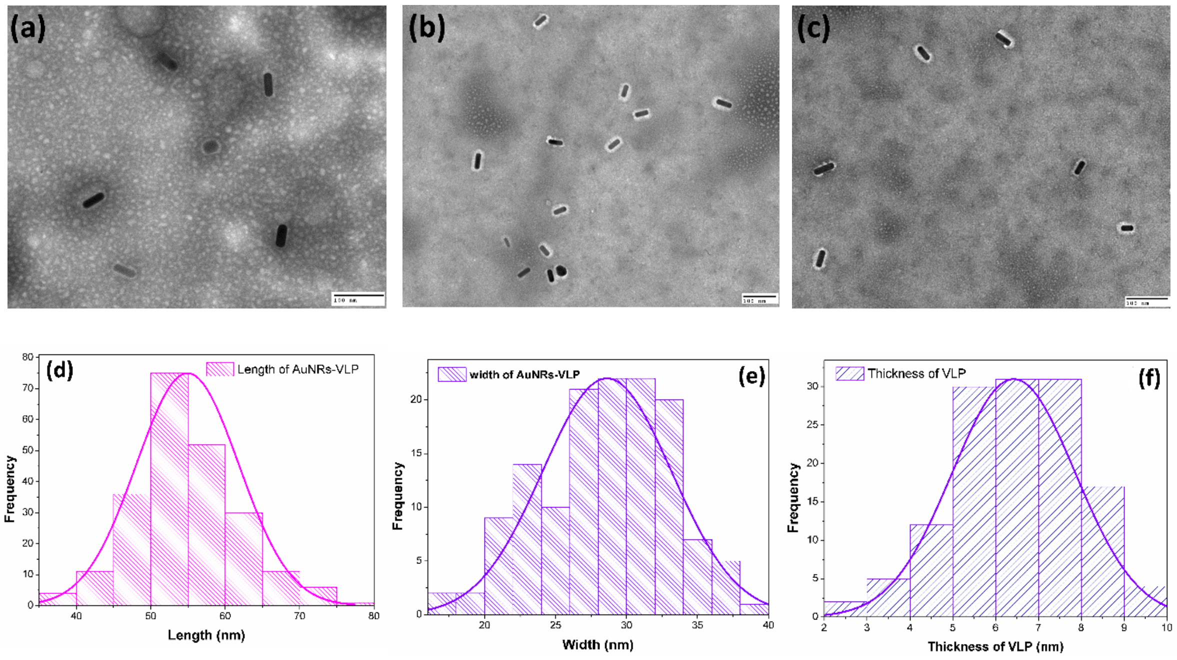

S6. TEM images of encapsidated AuNRs by the capsid protein of the CCMV virus a), b) and c), the scale bar in each image is 100 nm. d). Length distribution of AuNRs-VLP e). Width distribution of AuNRs-VLP and f). Thickness of VLP analyzing 100 AuNRs in two positions for each graph.
